# Suppression of Glioma Progression by Egln3

**DOI:** 10.1371/journal.pone.0040053

**Published:** 2012-08-08

**Authors:** Vicki A. Sciorra, Michael A. Sanchez, Akemi Kunibe, Andrew E. Wurmser

**Affiliations:** Department of Molecular and Cell Biology, Division of Cell and Developmental Biology, University of California, Berkeley, California, United States of America; Universidade de São Paulo, Brazil

## Abstract

Grade IV astrocytoma or glioblastoma has a poor clinical outcome that can be linked to hypoxia, invasiveness and active vascular remodeling. It has recently been suggested that hypoxia-inducible factors, Hifs, increase glioma growth and aggressiveness [1], [2], [3]. Here, we tested the hypothesis that Egl 9 homolog 3 (Egln3), a prolyl-hydroxylase that promotes Hif degradation, suppresses tumor progression of human and rodent glioma models. Through intracranial tumorigenesis and *in vitro* assays, we demonstrate for the first time that Egln3 was sufficient to decrease the kinetics of tumor progression and increase survival. We also find that Klf5, a transcription factor important to vascular remodeling, was regulated by hypoxia in glioma. An analysis of the tumor vasculature revealed that elevated Egln3 normalized glioma capillary architecture, consistent with a role for Egln3 in eliciting decreases in the production of Hif-regulated, angiogenic factors. We also find that the hydroxylase-deficient mutant, Egln3^H196A^ partially maintained tumor suppressive activity. These results highlight a bifurcation of Egln3 signaling and suggest that Egln3 has a non-hydroxylase-dependent function in glioma. We conclude that Egln3 is a critical determinant of glioma formation and tumor vascular functionality.

## Introduction

Glioblastoma is a highly invasive, fast-growing cancer, classified by hypoxia, necrosis and the active formation of intra-tumor blood vessels [4]. Collectively, the pathological features of glioblastoma render this malignancy extremely refractory to surgical resection, chemotherapy, radiotherapy and anti-angiogenic treatments, leading to a median patient survival of 12–15 months after diagnosis [5], [6], [7].

Many of the cellular and systemic adaptations to hypoxia (<8% O_2_) are mediated by hypoxia-inducible factors (Hifs) such as Hif-1α and Hif-2α, basic helix-loop-helix PAS domain transcription factors that regulate the expression of genes involved in angiogenesis, cell proliferation and metabolism [8], [9], [10]. For a variety of tumor types, high levels of Hif-1α and Hif-2α are tightly correlated with malignancy, invasiveness, metastasis and vascular density [10], [11], [12], [13], [14], [15], . While Hif-1α is ubiquitously expressed under hypoxia, Hif-2α exhibits a relatively restricted, cell type-specific pattern of expression [8], [17]. Hif-2α promotes tumor-initiation, the up-regulation of pro-angiogenic factors such as Vegf and expression of the embryonic stem cell gene, Oct4 [2], [18], [19], [20], [21], [22]. These data highlight Hifs as potential targets for dismantling the initiation and vascularization potential of glioma and raises the possibility that endogenously occurring Hif inhibitors could be employed to antagonize tumor progression.

Hifs are primed for degradation by Egln1, 2, and 3 hydroxylases (also termed prolyl-hydroxylase domain enzymes, PHD) that act upon specific proline residues in an O_2_-dependent manner and thereby target Hif-α subunits for ubiquitination by von Hippel-Lindau tumor suppressor and proteasomal-degradation [8], [23], [24]. Eglns manifest substrate biases in a number of cell contexts, with Egln1 prone to recognize Hif-1α and Egln3 preferentially hydroxylating Hif-2α [25]. Nonetheless, the roles of Egln1-3 in cancer biology are poorly understood, in some cases characterized as tumor suppressors and in others implicated in tumor aggressiveness [26], [27], [28], [29], [30], [31], [32]. Indeed, Egln proteins are heterogeneously expressed in various glioma cell lines *in vitro* [26], [30], making it difficult to correlate Egln expression with tumorigenicity. These ambiguities could be attributed to non-Hif related functions, as alternate Egln targets have been identified [26], [33], [34].

In this study, we report for the first time the effects of Egln3 expression upon glioma progression *in vivo*. When glioma-forming cells were engrafted intracranially and induced to express Egln3, tumor aggressiveness decreased as evidenced by markedly increased survival of injected mice. Using a catalytically inactive Egln3 mutant, we demonstrate that these effects are hydroxylase activity-dependent and independent, indicative of Hif and non-Hif functions of Egln3.

## Results

### Reduced Egln3 expression correlates with the up-regulation of Hifs in glioma cells

Hifs participate in maintaining the transcription of Oct4, a gene widely expressed within glioma cells, and are causal to aggressive glioma growth and progression [1], [2], [3], [18], [19], [20], [21], [22]. We therefore evaluated whether the expression of any member of the Egln prolyl-hydroxylase family correlated with the Hif-expression pattern during the hypoxic response of glioma cells. Since it previously has been reported that glioma cells differentially expressed Hif-2α relative to neural stem cells (NSCs) under hypoxia [2], we utilized NSCs as a control in these experiments.

**Figure 1:**
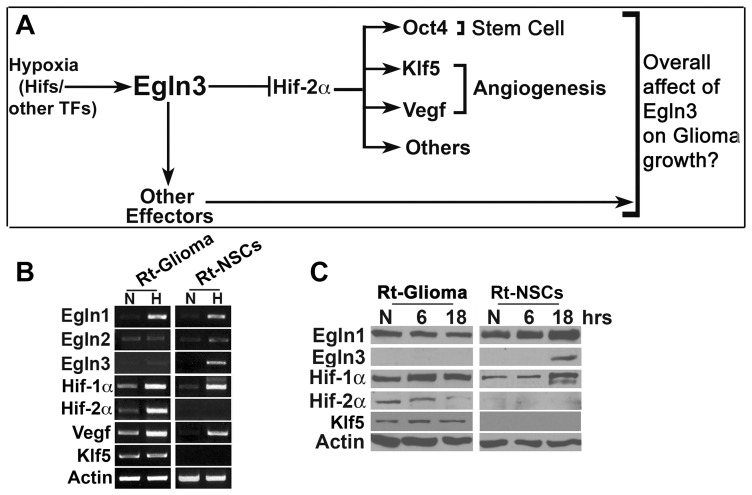
Hypoxic expression profile using a Rat glioma cell model. (A) Model for Egln3 function in glioma. (B, C) Rt-glioma cells or Rt-NSCs were subjected either to atmospheric O_2_ levels, normoxia (N) or to 1% O_2_, hypoxia (H) for the indicated time. mRNA (B) and protein (C) expression analysis of Egln1 Egln3c, Hif-1α, Hif-2α and Klf5 by RT-PCR and Western. β-actin served as a loading control.

We first compared the expression profiles of several hypoxia-response pathway components (Figure 1A) in Rt-glioma (F98; Rt-glioma) cells to Rat NSCs (Rt-NSCs) by RT-PCR (Figure 1B) and Western (Figure 1C). Egln1 transcript levels increased significantly under hypoxia (1% O_2_), consistent with reports indicating that Egln1 is hypoxia-responsive (Figure 1*B*) [30]. Egln2 mRNA was neither highly nor differentially expressed in Rt-glioma or Rt-NSCs, despite the use of several primer sets to probe for this transcript (Figure 1*B*; data not shown). On the other hand, Egln3 protein was undetectable in Rt-glioma cells (normoxia and hypoxia) whereas Egln3 in Rt-NSCs exhibited a dramatic increase upon hypoxia induction (Figure 1*B, C*).

Rt-glioma cells and Rt-NSCs each expressed Hif-1α (mRNA and protein, Figure 1*B–C*) and up-regulated the Hif gene target, Vegf, confirming that a hypoxic response had successfully been induced. In contrast, Rt-glioma cells, but not Rt-NSCs exhibited Hif-2αtranscript and protein (Figure 1*B–C*) [2]. Interestingly, Klf5 expression correlated with the relative absence of Egln3 and presence of Hif-2α, as Klf5 was not detected in Rt-NSCs and was up-regulated in Rt-glioma cells (Figure 1*B, C*). Klf5, a member of the Kruppel-like factor family of transcriptional regulators, encodes a key mediator of angiogenesis and arterial development [*35]*, [36].

**Figure 2:**
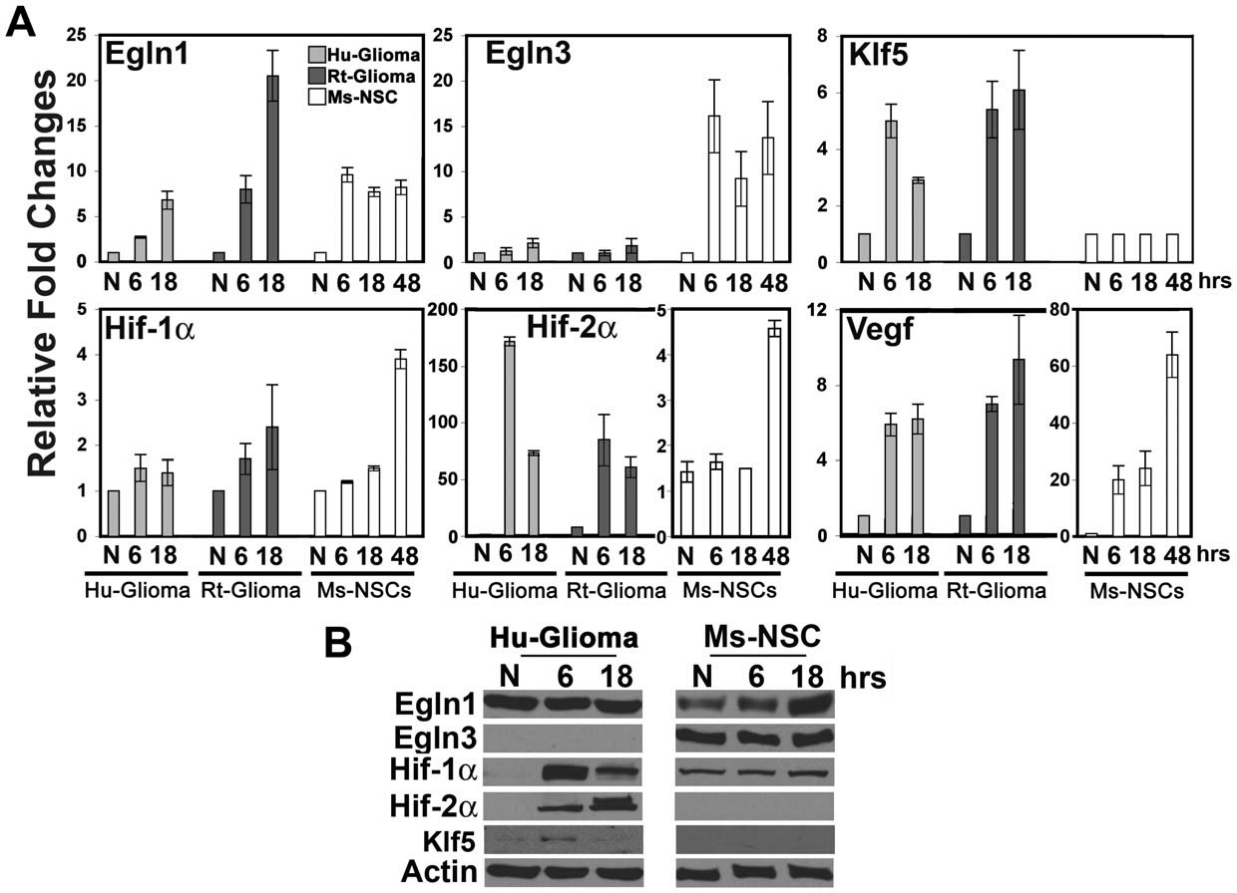
Hypoxic expression profile using an Human glioma cell model. Hu-glioma cells, Rt-glioma cells and Ms-NSCs were cultured under normoxic conditions and subjected to hypoxia for 6–48hrs, as indicated. (A) RT-QPCR was performed with primers specific for Egln1, Egln3, Klf5, Hif-1α, Hif-2α and Vegf. Data are expressed as fold changes relative to the normoxic condition for each respective cell type and normalized to β-actin mRNA. Mean values +/− s.d. are shown; n = 3. (B) Protein expression analysis of Hu-glioma cells and Ms-NSCs, as described in Figure 1.

We next examined whether this hypoxic gene response profile was conserved within human glioma (U87; Hu-glioma) cells, using mouse NSCs (Ms-NSCs) and Rt-glioma cells as controls. RT-QPCR (Figure 2A) and Western (Fure 2B) confirmed the absence of Egln3 expression in Hu-glioma cells and a hypoxia-dependent increase in Ms-NSCs. Similar to Rt-glioma cells, Hu-glioma cells upregulated both Hif-2αand Klf5 upon hypoxic insult (Figure 2A, B). These data suggest that Klf5 may be downstream of Hif-2αin glioma cells, as observed in liver hemangioma cells [9].

**Figure 3:**
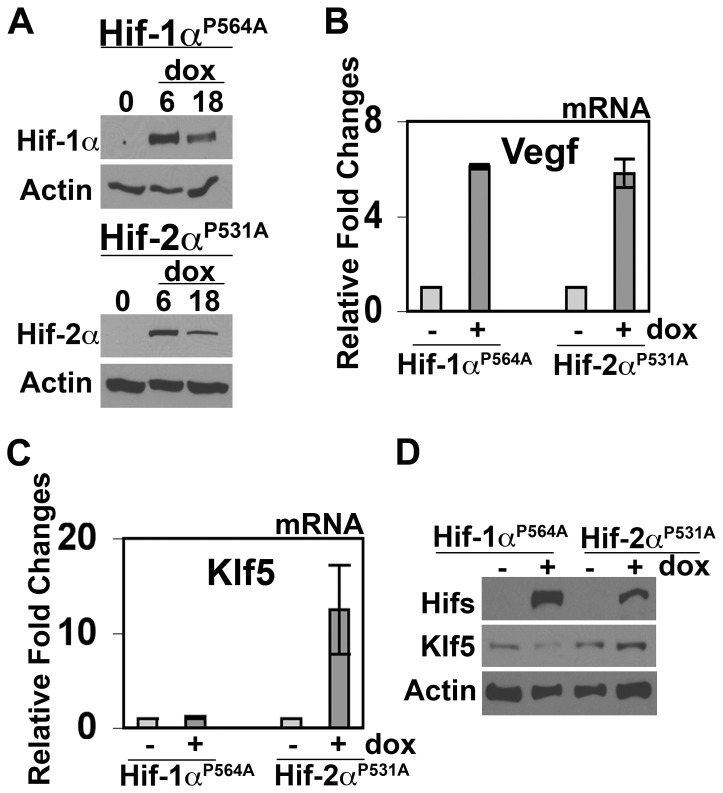
Hif-2αinduces Klf5-expression by glioma cells. (A) Normoxic Rt-glioma cells +/− 1µg/mL Dox treatment for 0–18hrs were analyzed by Western for the expression of degradation-resistant Hif-1α^P564A^ and Hif-2α^P531A^. (B, C) Normoxic Rt-glioma cells were induced with 1µg/ml Dox for 6hrs or 18hrs and analyzed for Vegf (B) or Klf5 (C) mRNA expression by RT-QPCR, respectively. Data are expressed as fold changes relative to no Dox controls. Mean values +/− s.d. are shown; n = 3. (D) Normoxic Rt-glioma cells were induced with 1µg/ml Dox for 18hrs and Klf5 protein expression assessed by Western.

In order to determine whether the expression of Hif-2αinfluenced the expression of Klf5 in glioma cells, we transduced Rt-glioma cells with an inducibly-expressed variant of Hif-2α(Hif-2α^P531A^) that is degradation-resistant under normoxia (Figure 3A) [37]. Induced expression of Hif-2α^P531A^ up-regulated Klf5 in Rt-glioma cells under normoxic conditions (Figure 3C, D). In contrast, an analogous Hif-1α^P564A^ mutant did not stimulate Klf5 (Figure 3C, D), even though Hif-1α^P564A^ and Hif-2α^P531A^ constructs were comparably expressed and each sufficient to induce Vegf (Figure 3A, B).

**Figure 4:**
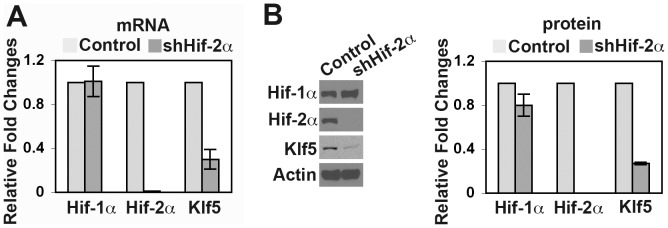
Suppression of Hif-2α reduces Klf5 expression in Hu-glioma cells. Hu-glioma cells expressing luciferase shRNA (control) or Hif-2α shRNA (shHif-2α) were subjected to hypoxia for 6hrs and analyzed for gene expression by RT-QPCR (A) and Western (B). Data are expressed as fold changes relative to shRNA controls.

To provide additional evidence that Klf5 can respond downstream of Hif-2α in glioma cells, we expressed a shRNA designed to specifically silence Hif-2α [38] in Hu-glioma cells. shHif-2α severely curtailed the expression of Hif-2αtranscript and protein (Figure 4) to nearly undetectable levels in Hu-glioma cells under hypoxia, while not significantly affecting Hif-1αConsistent with the hypothesis that Klf5 acts downstream of Hif-2α, knockdown of Hif-2αin Hu-glioma cells resulted in ≈70% reduced expression of Klf5 (Figure 4). Collectively, these data suggest that Klf5 expression can serve as a downstream readout for Hif-2αactivity (Figure 1A). Our results also raise the possibility that Klf5 mediates certain Hif-dependent downstream functions within glioma.

### Egln3 suppresses Hif-2α, Oct4 and Klf5 expression within cultured glioma cells

Since the relative absence of Egln3 correlated with Hif-2α expression in glioma cells, we predicted that the expression of Egln3 might be sufficient to influence the hypoxic response of these cells. To assess this possibility, we inducibly-expressed Egln3 and assayed for effects in the Hif expression pattern of glioma cells.

**Figure 5:**
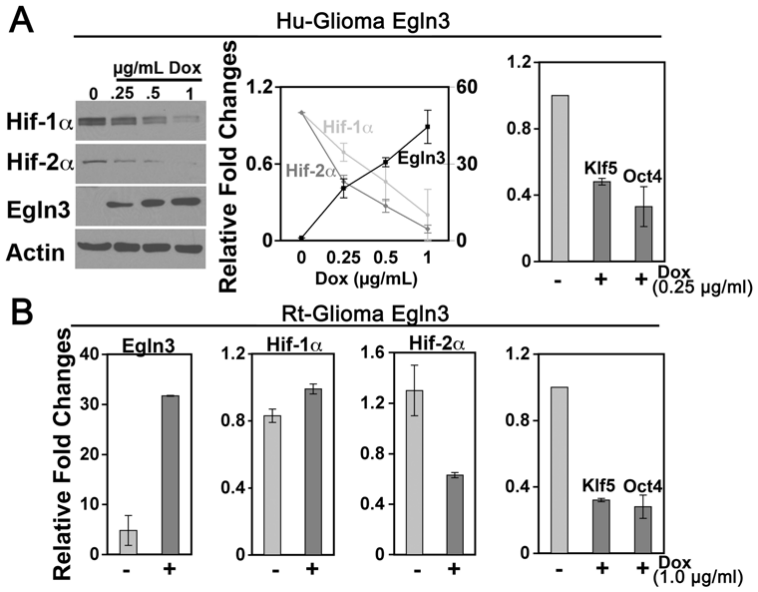
Inducibly-expressed Egln3 down-regulates Hif-2α, Oct4 and Klf5 in glioma cells. (A) Total cell lysates were prepared from Hu-glioma cells grown for 6hrs under hypoxia in the presence of 0–1µg/ml of Dox and analyzed for Egln3, Hif-1α and Hif-2α expression by Western. Changes in protein expression levels of Hif-1α ℓ (y-axis scale, 0–1.2), Hif-2 _ (y-axis scale, 0–1.2), Egln3 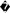 (y-axis scale, 0–60) were quantified relative to controls (hypoxia 6hrs, -Dox). Klf5 and Oct4 mRNA expression levels were determined by RT-QPCR (far right panel) performed on Hu-glioma cells Egln3 +/− 0.25µg/mL Dox that were cultured under hypoxia for 6hrs. (B) Rt-glioma cells Egln3 +/− 1µg/ml Dox were assayed for Egln3, Hif-1α and Hif-2α expression by Western. Klf5 and Oct4 mRNA expression analysis was conducted by RT-QPCR (far right panel). Hypoxic samples are shown; data are expressed as fold changes relative to control (hypoxia 6hrs, -Dox). Mean values +/− s.d. are shown; n = 3.

Hu-glioma cells exhibited a dosage-dependent induction of Egln3 with doxycycline (Dox) levels of 0–1µg/mL. 2–4 fold elevation of Egln3 protein expression was achieved compared to Ms-NSCs over this Dox concentration range. Relative to Hif-1α, Hif-2α protein levels were sensitive to Egln3 after a 6hr treatment with 0.25µg/mL Dox under hypoxia whereas higher induction levels (1µg/mL Dox) destabilized both Hif-1α and Hif-2α (Figure 5A). This analysis was necessary to determine a working concentration of Dox to evaluate the functional significance of Egln3 within glioma cells. Low levels of Egln3 induction (0.25µg/mL Dox) reduced the transcription of Klf5 and the Hif-2α transcriptional target, Oct4 [18] by ≈50% and ≈70%, respectively within Hu-glioma cells relative to hypoxia-treated controls (Figure 5*A*). Similarly, for Rt-glioma cells infected with a Dox-inducible Egln3 construct, the presence of 1µg/mL Dox (6hrs) up-regulated the expression of Egln3 protein and caused ≈50% decreases in Hif-2α protein levels after a 6hr exposure to hypoxic conditions without significantly altering Hif-1α expression (Figure 5B). The increased expression of Egln3 coincided with ≈75% decreases in Klf5 and Oct4 (Figure 5B). Therefore, the introduction of Egln3 into Hu- and Rt-glioma cells was biased to uncouple the hypoxia-initiated adaptive responses mediated by Hif-2α relative to Hif-1α.

### Egln3 decreases glioma progression in vivo

**Figure 6:**
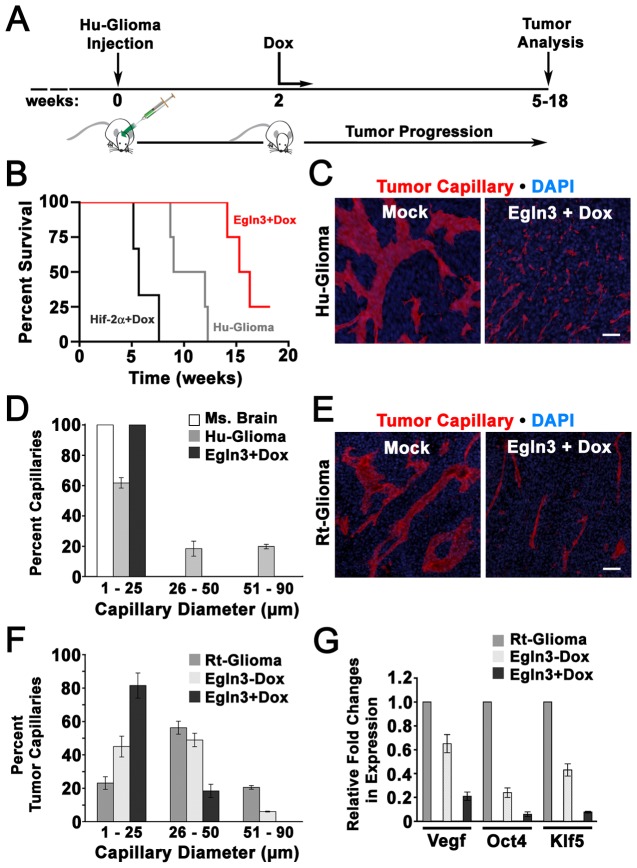
Expression of Egln3 decreases tumor aggression, increases survival and normalizes glioma vascular architecture. (A) Timeline for the intracranial xenoengraftment of Hu-glioma initiating cells, Dox-induced expression of Egln3 in tumor cells and tumor analysis. (B) Kaplan-Meier survival curves for Hu-glioma (empty vector transfected; grey), Hu-glioma Egln3 + Dox (red), and Hu-glioma Hif-2α^P531A^ + Dox as a control (black); in total, n = 18). (C) Fluorescent images of intra-tumor blood capillaries. Gliomas originating from Hu-glioma initiating cells (empty vector transfected) and Hu-glioma Egln3 + Dox cells were allowed to develop until the onset of neurological symptoms and labeled with anti-CD31 (PECAM) antibodies and DAPI. Scale Bar  = 100µm. (D) Percentage of CD31-labeled blood capillaries that exhibited diameters of 1–25µm, 26–50µm or 51–90µm relative to internal scale bars in non-cancerous adult mouse brain, Hu-gliomas (empty vector transfected) and Hu-gliomas Egln3 + Dox after tumor progression elicited neurological symptoms. >500 capillaries of 10 randomly chosen tumor sections were chosen from 3 glioma engrafted mice for each experimental group. Mean values +/− s.d. are shown; n = 4. (E) Rt-glioma (empty vector transfected) and Rt-glioma Egln3 + Dox were allowed to develop until the onset of neurological symptoms and fluorescently labeled with the endothelial-specific probe, isolectin GS-IB_4_ (Lectin) and DAPI. Scale Bar  = 100µm. (F) Percentage of Lectin-labeled tumor blood capillaries that exhibited diameters of 1–25µm, 26–50µm or 51–90µm in empty vector transfected Rt-gliomas and Rt-gliomas Egln3 +/− Dox after tumor progression elicited neurological symptoms. Mean values +/− s.d. are shown; n = 4. (G) Empty vector transfected Rt-glioma and Rt-glioma Egln3 +/− Dox tumors were allowed to progress until neurological symptoms were noted. Quantification of Vegf, Oct4 and Klf5 mRNA expression in total tumor were determined by RT-QPCR. Data are expressed as fold changes relative to empty vector transfected Rt-glioma tumor samples. Mean values +/− s.d. are shown.

Based upon a critical role for Hifs in promoting tumor progression [10], [11], [12], [13], [14], [15], [16], it was reasonable to speculate that the induction of Egln3 might attenuate glioma growth. To determine if Egln3 could influence the kinetics of glioma development *in vivo*, we stereotaxically-engrafted Hu-glioma cells expressing Egln3 under the control of a Dox-inducible promoter into the cerebral cortex of immuno-compromised (NSG) mice. After 2 weeks of engraftment, mice were fed Dox daily and the experiment terminated upon the initial manifestation of neurological symptoms, an indicator of glioma formation (Figure 6A). While mock-treated Hu-gliomas developed within 9–12 weeks, Hu-gliomas induced to express Egln3 did so consistently between 14–16 weeks, as represented by a Kaplan-Meier plot in Figure 6B. In contrast with mock-treated Hu-glioma cells that always initiated tumor formation, a single animal injected with Hu-glioma Egln3 +Dox failed to develop neurological symptoms after a ≈18 week period (Figure 6B). Microscopic analysis of the engraftment site of this Hu-glioma Egln3 +Dox animal confirmed that tumor formation had failed to occur.

To further validate our assay we inducibly-expressed Hif-2α^P531A^, a pro-tumorigenic factor [2], [38], within Hu-glioma. When Dox-administration was started 2 weeks after Hu-glioma Hif-2α^P531A^ engraftment, tumor growth accelerated, resulting in experiment termination between 5–8 weeks (Figure 6B). Therefore, induction of Egln3 two weeks following engraftment significantly reduced overall Hu-glioma aggressiveness.

### Egln3 promotes normalization of glioma capillary morphology in vivo

Since Egln3 was sufficient to induce decreases in Hif signaling *in vitro* (Figure 5), the expression of Egln3 might also influence blood vessel formation within glioma. To test this possibility, we assessed CD31 (PECAM-1)-labeled blood capillaries within Hu-gliomas that were allowed to progress until neurological symptoms developed, facilitating an analysis of tumors of comparable size. As is typical of the inefficient blood vessels of solid tumors such as glioblastoma [39], [40], [41], the vascular architecture of Hu-glioma tumors were distended and haphazardly organized, with a significant percentage of capillary branches exhibiting diameters between 26–50µm (≈20%) or capillary diameters of >50µm (≈20%). In contrast, non-tumor capillaries of adult mouse brain had diameters of 1-to-25µm (Figure 6C, D). Strikingly, induction of Egln3 normalized tumor capillary morphology, reducing the percentage of disorganized and distended vascular branches (0% tumor capillaries >25µm in diameter; Figures 6C, D).

Utilizing an endothelial cell-specific probe, isolectin GS-IB_4_ (Lectin) [42] to label intra-tumor Rt-glioma capillaries, we found that Rt-gliomas also exhibited significant percentages of capillaries with exaggerated diameters relative to healthy brain tissue (≈55%, 26–50µm and ≈20%, 51–90µm; Figure 4E, F). Egln3 up-regulation evoked a relatively normalized tumor capillary structure with 80% of capillaries having diameters of <26µm, 20% with diameters of 26–50µm and 0% of >50µm (Figure 6E, F).

### Egln3 suppresses the expression of Klf5 and the Hif transcriptional targets, Oct4 and Vegf in vivo

Consistent with our *in vitro* experiments, a quantitative analysis of total tumor transcript levels indicated that Dox-induced Egln3 reduced Vegf by ≈80%, Klf5 by ≈90% and Oct4 by ≈90% in Rt-gliomas (Figure 6G). Intermediate reductions in the expression of these genes were achieved in Rt-glioma Egln3 -Dox tumors due to a basal level of Egln3 expression by our construct (data not shown). Therefore, Egln3 decreased the expression of Klf5 and several hypoxia-regulated targets *in vivo*.

### Catalytically-inactive Egln3^H196A^ partially suppresses glioma growth

**Figure 7:**
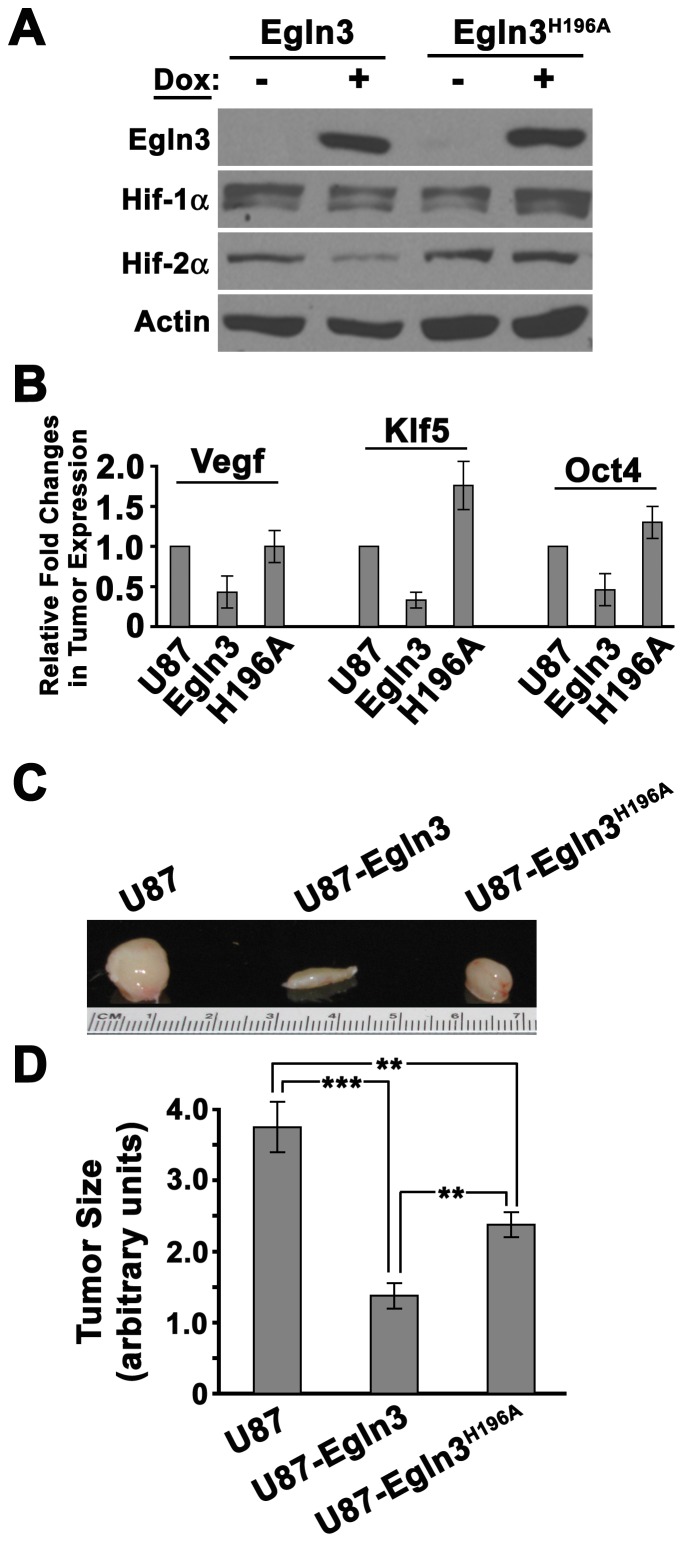
Hydroxylase- dependent and independent roles of Egln3 in glioma progression. (A) Western analysis of Hu-glioma cells induced to express wild-type Egln3 or an hydroxylase defective mutant, Egln3^H196A^ with Dox (0.25µg/mL) for 6hrs under hypoxia *in vitro*. (B, C, D) Mock-treated Hu-glioma cells or Hu-glioma transfected with Dox-inducible Egln3 or Egln3^H196A^ constructs were injected subcutaneously into the flanks of NSG mice. Dox was administered once daily beginning 1 week following engraftment. Mean values +/– s.d. are shown; n = 3. (B) Quantification of mRNA expression in total tumor samples were determined by RT-QPCR. (C, D) Expression of Egln3 or the hydroxylase-defective Egln3^H196A^ decreased glioma progression. Tumor mass was determined 30 days post injection. The asterisks (**) or (***) indicates significance with a *p* value of ≤0.001 or ≤0.0001, respectively.

While many of the known effects of Egln family members are mediated through the hydroxylation of Hifs and possibly other targets, catalytically inactive Egln1 has been shown to maintain hydroxylase-independent functions through additional signaling interactions [26]. To address whether the tumor suppressive phenotype of Egln3 was intrinsically linked to hydroxylase activity, we generated an enzymatically defective Egln3 mutant through the alteration of a catalytically critical histidine residue (i.e. H196A) [26], [34], [43]. Consistent with previous studies [43], Egln3^H196A^ was expressed at levels comparable to wild-type Egln3 in Hu-glioma cells *in vitro* and did not promote the degradation of Hif-1α or Hif-2α (Figure 7A). In addition, quantification of total tumor Vegf, Klf5 and Oct4 transcript levels indicated that Egln3^H196A^ induction did not inhibit Hif-mediated transcription whereas tumors expressing wild-type Egln3 displayed decreases in Vegf, Klf5 and Oct4 *in vivo* (Figure 7B). In fact, Egln3^H196A^ evoked increases in both Klf5 and Oct4 that we speculate may be due to potential dominant-negative effects of Egln3^H196A^ expression within glioma upon other signaling pathways (e.g. Egln1).

We next ascertained whether Egln3^H196A^ affected the kinetics of tumor progression by injecting Hu-glioma cells expressing Egln3 or Egln3^H196A^ under the control of a Dox-inducible promoter into the flanks of NSG mice. One week following engraftment, mice were fed Dox daily and the experiment terminated after 30 days. Consistent with our intracranial engraftment assays (Figure 6), Egln3 expression dramatically reduced tumor size (Figure 7C, D). Intriguingly, the hydroxylase-deficient Egln3^H196A^, exhibited tumor growth intermediate to mock-treated and Egln3-expressing human gliomas. Collectively, these data confirm a key role for Egln3 in glioma progression through the hydroxylation of Hifs and through the participation of Egln3 in non-hydroxylase-dependent signaling pathways (see Discussion).

## Discussion

### Egln3 is sufficient to attenuate glioma progression

In this study, we have assessed the effects of Egln3 upon the development of glioma *in vivo*. According to The REMBRANT Database, some human gliomas exhibit a highly aggressive, low Egln3-expression phenotype (i.e. >2-fold decreases in Egln3 relative to basal brain levels), analogous to Hu- and Rt-gliomas in which Egln3 protein was not detected. This raised the possibility that the down-regulation of Egln3 might contribute to the progression and aggressiveness of some gliomas by accentuating the activity of Hif-mediated signaling within glioma cells. Given the role of Hifs in the progression of a variety of tumor types, Eglns could be promising candidate suppressors of tumor growth. Further experimentation demonstrated that Eglns influence other cellular processes as well, since several non-Hif targets of Eglns have been identified including cyclin D1, NF-kappaβ and the β2-adrenergic receptor [26], [33], [34]. More recently, the Egln1 hydroxylase has been shown to have a Hif-independent function on amphiregulin regulation in breast carcinoma [44]. Our data suggest that Egln3 follows this precedent within glioma as well. Therefore, the interfacing of Eglns with Hif and non-Hif factors appears to influence tumorigenesis, highlighting the need to mechanistically dissect Egln3 signaling pathways in glioma and necessitating an analysis of the overall effects of Eglns upon the progression of gliomas and other malignant tumor types.

We hypothesize that induction of Egln3 during the initial stages of tumor formation impaired cellular factors that are proposed to support tumor initiation such as Hif-2α and Oct4 [2], [19] and that later stage glioma progression was abrogated through reduced angiogenic signaling within the tumor. Within cultured glioma cells it has been suggested that Egln3 can support cell viability, by attenuating exaggerated Hif signaling [30]. The dosage and timing of Egln3 induction as well as the targeted expression of Egln3 to specific glioma cell types likely will prove to be important considerations in directing the tumor suppressing potential of Egln3. Therefore, while the overall effect of Egln3 was to suppress Hu-glioma growth, our findings also raise the prospect of additional Egln3 effectors within glioma.

### Egln3 as a vascular normalization factor within glioma

Relative to healthy tissue, many solid tumor types including glioblastoma are believed to overproduce pro-angiogenic factors, resulting in the distension and disorganization of tumor vasculature [39], [40], [41]. We noted that Egln3 reduced tumor Vegf production and induced the normalization of capillary structure. Vascular normalization through the administration of angiogenic inhibitors has arisen as a promising means of enhancing the circulation and efficacy of chemo- and radio-therapeutics [45], [46]. Interestingly, it has been hypothesized that angiogenesis inhibitors can be dosed to simultaneously decrease capillary number and normalize vessel structure, avoiding increased tumor growth kinetics [45]. According to this hypothesis, our observed decreases in tumor aggression upon Egln3 induction would be consistent with depressed levels of angiogenesis within Egln3-expressing Hu-gliomas. Identifying cellular signaling factors that can be modulated to elicit vascular normalization has therefore become of great interest [45]. Nitric oxide and Rgs5 have been revealed as key regulators [47], [48] and the haplodeficiency of Egln1 within host mice (i.e. not within engrafted tumor cells) also influenced intra-tumor capillary structure [31]. We propose that Egln3 encodes an additional, molecular participant that governs tumor vascular normalization. Clearly Egln3 participates as a regulator of many fundamental cellular processes, highlighting the complex nature of Egln3 function in glioma formation.

## Materials and Methods

### Cell culture

Human U87 (Hu-glioma) [49] and rat F98 (Rt-glioma; ATCC, Manassas, VA) cells were grown in RPMI (Mediatech, Inc., Manassas, VA) supplemented with 10% fetal bovine serum and L-glutamine (Omega Scientific, Inc., Tarzana, CA) or in neural-basal serum-free media [42], [50]. Hypoxic stress experiments were conducted under atmospheric O_2_ levels (normoxia; N) or 1% O_2_ (hypoxia; H) for the indicated time period (6–48hrs). Primary mouse [42] and rat neural stem cells (NSCs) [51] were isolated and cultured as previously reported [52].

Egln3, Hif-1α and Hif-2α retrovirus were generated using 293T retroviral packaging cells [51] transfected by calcium phosphate. Hu- and Rt-glioma cells were infected with rtTA, tetracycline-responsive transactivator (Clontech, Mountain View, CA) and selected 72 hrs using 400µg/mL G418 (MP Biomedicals, Solon, OH). G418 resistant cell populations were subsequently infected with inducible, Egln3 retrovirus. Egln3 infected cells were selected for 72 hrs using 50µg/mL hygromycin (Omega Scientific, Inc.).

### Transcript expression analysis

RT-PCR was performed as described [42] with primers specific for each gene. Glioma RNA was prepared by resecting the tumor, dounce homogonizing the tissue and purifying total RNA using RNeasy Plus Mini Kit (Qiagen, Valencia, CA). Relative mRNA levels of Egln1, Egln3, Hif-1α, Hif-2α, Vegf, Oct4 and Klf5 were determined by reverse transcription-quantitative polymerase chain reaction (RT-QPCR), as described [2] using GoScript Reverse Transcriptase (Promega Corp,). Fold changes were calculated as a percentage relative to the relevant corresponding control after normalized to actin.

### Protein expression analysis

Cells were harvested in PBS containing 100 µM PMSF, 1 mM DTT, 50 mM NaF, 200 µM Na_3_VO_4_, a protease inhibitor cocktail (EMD Chemicals, Germany) and boiled in Laemmli buffer. Antibodies specific to Hif-1α (Millipore, Temecula, CA), Hif-2α, Egln1, Egln3 (Novus Biologicals, Littleton, CO; Santa Cruz Biotech., Santa Cruz, CA) and Klf5 (Millipore) were used. Staining with β-actin antibody (Abcam, Cambridge, MA) served as a loading control. Protein bands were visualized by enhanced chemiluminescence using Super Signal (Pierce, Rockford, IL). Relative protein levels were calculated by using ImageJ (National Institute of Health, MD) and Adobe Photoshop (Adobe Systems, San Jose, CA) software using Actin as a normailization standard.

### DNA manipulations

The cDNA encoding Hif-1α (NM_001430) was provided by Gregg Semenza, Johns Hopkins University School of Medicine, Baltimore, MD. Degradation-resistant variants of human Hif-1α and human Hif-2α (cloned from a human endothelial cell cDNA library; NM_001530) were generated by altering proline^564^ or proline^531^ to alanine by site-directed mutagenesis of Hif-1α and Hif-2α, respectively. The coding region for Egln3 (NM_028133) was isolated from a mouse NSC cDNA library by PCR. The hydroxylase-deficient variant of mouse Egln3 was generated by altering histadine^196^ to alanine by site-directed mutagenesis. The sequences of all Hif and Egln3 constructs were verified. The mutated Hifs and Egln3 were cloned into a Dox-inducible retroviral vector, pTre-tight modified to include the hygromycin resistance gene (Clontech, Mountain View, CA) and introduced into glioma cells.

Human Hif-2α shRNA sequences were expressed using plasmid 22101: pRS9 Hif-2α-pRetro-Super [38] (Addgene Inc., Cambridge MA). Hu-glioma cells expressing this Hif-2α shRNA were drug-selected (750 ng/ml puromycin) as described above.

### Intracranial and subcutaneous injections of glioma cells

NSG mice (NOD.Cg-Prkdcscid Il2rgtm1Wjl/SzJ; The Jackson Laboratory, Bar Harbor, Maine) were positioned in a stereotaxic frame (David Kopf Instruments, Tujunga CA) and 100,000 Hu-glioma or 50,000 Rt-glioma cells in a volume of 1µL were intracranially engrafted into the cerebral cortex, as described [2]. 1mg doxycycline (Dox) was administered in food *ad libitum* per day for ≈1–3 weeks. For Hu- and Rt- gliomas this Dox regimen was started 2 weeks or 1 week following cell engraftment, respectively. Upon onset of neurological symptoms, mice were sacrificed for tumor analysis. For subcutaneous injections, 1×10^6^ Hu-glioma cells were mixed 1∶1 with growth factor reduced matrigel (BD Biosciences, Bedford, MA), injected in the flanks of NSG mice and dox was administered as described above starting 1 week following engraftment. All mouse procedures were carried out in accordance with applicable IACUC and federal guidelines and protocols were approved by the Animal Care and Use Committee of the University of California, Berkeley (Animal Use Protocol # R318-1011B).

### Microscopy

Images were obtained with an IX71 microscope system (Olympus America Inc, San Diego, CA)/Retiga 2000R cooled camera (QImaging, Surrey, BC Canada). Anti-CD31 antibody was used to label and assess Hu-glioma capillaries (BD Biosciences, San Jose, CA). Secondary antibodies coupled to the Alexa-555 dye (Invitrogen) was used as described [42]. Rt-glioma capillaries were labeled using and endothelial-specific isolectin GS-IB_4_ from *Griffonia simplicifolia* conjugated to Alexa-594 (Lectin; Invitrogen) at 1µg/ml, according to the manufacturers instructions. Nuclei were stained with 4′,6-Diamidino-2-phenylindole, dihydrochloride (DAPI; Anaspec, Fremont, CA). Tumor capillaries were delineated by labeling tumor sections with CD31 or EC Lectin. Capillary diameter was then quantified relative to scale bars to determine capillary diameter. For each experimental group, >500 capillaries of 10 randomly chosen tumor sections were chosen from 3 glioma engrafted mice.

### Statistical Analysis

Statistical analysis was performed using a standard two-tailed T test.
